# Characterization of blood microbial population in beef calves with clinical signs of sepsis using 16S rRNA gene sequencing

**DOI:** 10.1371/journal.pone.0324469

**Published:** 2025-05-23

**Authors:** Giuliano Borriello, Flaminia Valentini, Sara Ferrini, Giorgia Di Muro, Giulia Cagnotti, Elena Grego, Angela Maria Catania, Maria Cristina Stella, Ugo Ala, Patrizia Nebbia, Antonio D’Angelo, Claudio Bellino

**Affiliations:** Department of Veterinary Sciences, University of Turin, Italy; University of Minnesota, UNITED STATES OF AMERICA

## Abstract

Sepsis, a dysregulated host response to infection, severely affects calf health. To date, sepsis diagnosis relies on clinical examination and positive blood culture. Differently, in humans 16S rRNA gene analysis is a valuable adjunct to blood culture as it allows for broader assessment of bacterial DNA in whole blood and its fractions. However, its efficacy in cattle remains unknown. Therefore, this study characterized and compared the bacterial DNA detected in whole blood and its fractions between healthy calves and those showing clinical signs of sepsis. The study sample was 18 Piedmontese calves classified according to their clinical status as suspected septic (**S,** 8/18) or healthy (**H,** 10/18). Aseptic blood samples were collected into EDTA tubes for 16S rRNA gene analysis of whole blood (**WB**), plasma (**PL**), buffy coat (**BC**), and red blood cells (**RBC**). Aseptic samples were additionally taken only from the **S** calves for blood culture. Clinical and microbiological parameters were compared between the two groups and between the blood fractions within each group. The **S** calves were diagnosed with pneumonia (3/8, 37.5%), enteritis (3/8, 37.5%), and omphalitis (2/8, 25%). Microbiome analysis revealed significant intra-group differences in α and β diversity indices between **PL** and the other blood fractions for both groups. Comparison between the **S** and the **H** calves showed differences in β diversity indices for **PL** and **WB**. DNA of known pathogens (e.g., *Escherichia coli*) and species not commonly associated with sepsis (e.g., *Cutibacterium acnes*) were more abundant in the **S** calves. Moreover, in **S** calves, 16S rRNA gene sequencing detected *E. coli* DNA more often (8/8, 100%) than blood culture (2/8, 25%). While the DNA of several bacteria can be detected in calves showing clinical signs of sepsis, further studies are needed to clarify its origin, role, and distribution in blood fractions.

## Introduction

Calves are particularly susceptible to infection during their first weeks of life. In severe cases, a dysregulated host response can lead to life-threatening organ dysfunction, also known as sepsis [1]. Despite its impact on animal health, sepsis remains under-investigated in farm animals. Clinical diagnosis relies on the detection of severe clinical alterations along with bacterial assessment in the bloodstream by blood culture [1,2]. Such methods have limitations, however. Indeed, clinical parameters (e.g., respiratory and heart rates) can be influenced by the animal’s behavioral response to clinical examination, leading to potentially inaccurate assessment [3]. The scoring systems used in human medicine are more accurate but have not yet been validated in cattle [4]. Blood culture, although it is the gold standard, may have suboptimal sensitivity and specificity, especially in cases of slow-growing bacteria or low bacterial load [5,6]. Moreover, the risk of blood culture contamination seems to be higher for animals than for humans [7,8]. However, it remains unclear whether the higher risk is due to the environment, differences in the microbial skin population or the presence of hair surrounding the shaved area. In recent years, 16S rRNA gene sequencing has gained attention because of its ability to partially overcome the limitations of blood culture. Human studies have reported that it can detect traces of bacterial DNA in healthy and diseased subjects [9–11] and identify bacterial DNA in the blood of septic patients, even when blood cultures are negative [12]. Further, 16S rRNA gene sequencing has proved reliable for comprehensive analysis of the entire bacterial DNA within a sample and highlighted differences between the bacterial DNA found in blood of healthy and septic human patients [13]. In addition, it revealed differences in the microbiome of blood fractions in physiological and pathological conditions, even though the underlying reasons for these differences still need to be clarified [9,14,15]. In bovine species, 16S rRNA gene sequencing has been applied to characterize the microbiome of body districts in healthy and diseased animals [10,16–18]. Nevertheless, to our best knowledge, there are no studies on blood bacterial populations in septic calves. Understanding the characterization of bacterial populations by 16S rRNA gene sequencing could be the first step to comprehend the role that microorganisms play in sepsis. For these reasons, with the present study, we aimed to characterize and compare the bacterial DNA detected in whole blood and its fractions between healthy calves and those suspected septic.

## Materials and methods

### Study population

This analytical cross-sectional study was conducted from December 2022 to July 2023 and involved Piedmontese breed calves reared in northern Italy (Piedmont) referred for suspected sepsis or examined during routine herd visits. Calves were considered eligible if they were within the neonatal period (3 to 30 days old) [19] and had not been receiving antimicrobial treatment before or at the time of examination. All the procedures were conducted on the farms or at the Veterinary Teaching Hospital, University of Turin, in conformity with good clinical practices [20]; the study was approved by the Ethical Animal Care and Use Committee, University of Turin (Prot. n. 0004177). The owners were informed about the procedures, the potential risks of the study and signed an informed consent form for veterinary assessment of their animals.

### Clinical examination and hematobiochemical analysis

Medical history of each calf was collected on enrollment into the study. A complete clinical examination and blood sampling for hematobiochemical analysis to determine the clinical status were performed by the same operator (first author, GB). Blood was collected from the jugular vein with a 21G needle and Vacutainer^®^ (Becton and Dickinson, Franklin Lakes, NJ, USA) EDTA and serum tubes. The samples were analyzed at the Internal Medicine Unit, Department of Veterinary Sciences of Turin laboratory on an ADVIA® 120 (Siemens, Munich, Germany) for biochemical analysis and, for complete blood count (CBC) an BT3500Vet (Futurlab, Limena, Italy).

The calves were classified as critically ill if severe clinical alterations and at least one focal site of infection were found. When needed, further blood tests or other diagnostic techniques (i.e., ultrasonography, radiography) were performed, using appropriate anesthetic and analgesic protocols if needed, to identify the infectious focus in the CI calves. Then, each CI calf was classified based on the systemic inflammatory response syndrome (SIRS) score [21]. If a calf was classified as affected by the SIRS, the probability of bacteremia was estimated using the score described in Fecteau [22] (Table 1). Animals were classified as suspected septic (**S**) if the probability score was higher than 40.8%, as specified by the original author [22].

**Table 1:** Scoring sheet for scoring sepsis and predicting bacteremia.

Criteria	Results	Points
**Focal site of infection**	No	**0**
	Yes	**1.5**
**Age (Days)**	<7	**0**
	≥7	**1.2**
**Clinical Score**	**Qualifiers**	
	**FECAL**	
**0**	Normal feces	
**1**	Feces softer than normal but no diarrhea on tail	
**2**	Diarrhea but not profuse, wet tail	
**3**	Profuse watery diarrhea, wet tail, soiled pen or indication of blood or fibrin in feces	
	**HYDRATION**	
**0**	Normal if skin tent <2 s	
**1**	Moderate dehydration if eyeball slightly sunken, skin tent between 2 and 4 s	
**2**	Obvious dehydration if eyes sunken, dry nose, skin tent >5 s	
**3**	Severe dehydration if eyes very sunken with an easily perceptible distance between the eyeball and the eyelid, skin tent persistent	
	**ATTITUDE**	
**0**	Normal behavior, alert, gets up when approached, interested in surroundings	
**1**	Depressed, must be stimulated to get up	
**2**	Gets up only with help	
**3**	Unable to stand, even with help	
	**UMBILICUS**	
**0**	Normal if pencil size, dry and painless	
**1**	Bigger than normal but dry and painless	
**2**	Bigger than normal, wet or painful	
**3**	Bigger than normal, with pus draining and evidence of pain (presence of internal umbilical swelling ranks as 3 and should be described as involving arteries/urachus or vein)	
	**SCLERAL VESSEL**	
**0**	Normal (<2), does not reach the limbus	
**1**	Increased in number (<4), at least 1 reaches the limbus, color is still pink, size is normal	
**2**	Increased in number, between 4 and 6, at least 2 reach the limbus, color is red, size is mildly increased	
**3**	Increased in number (>6), at least 3 reach the limbus, color is purple, size is greatly increased	
**Sum**	≤5	**0**
	>5 and ≤8	**2.1**
	> 8	**2.5**

Conversely, calves with no clinical signs were enrolled in the healthy (**H**) control group. Whenever possible, the **H** calves were selected from the same farm as the **S** calves, matched for age and sex. If this was not feasible, **H** calves were enrolled from nearby farms where an **S** calf was involved, ensuring that the managerial procedures and the environmental conditions were similar.

### Sterile blood sampling

Sterile blood samples were collected from the **S** and the **H** calves. For each calf, the hairs from the jugular groove were completely removed using an electronic shaver and a handled razor. The skin was then scrubbed as described by Garcia [23] to ensure asepsis of the sampling site. Blood samples were collected with a 21G needle into two Vacutainer® EDTA tubes for 16S rRNA gene analysis. The samples were refrigerated at 4° C during transport. An additional aseptic sampling for blood culture was performed only for the **S** calves. To minimize the risk of contamination, the scrub was repeated and blood was drawn using a 21G needle and a 10-ml syringe. The blood was immediately added to a culture bottle containing enriched media (Oxoid Signal^®^ Blood Culture System. Oxoid Limited, Basingstoke, UK). All the samples were delivered to the laboratories of the Infectious Disease Unit, Department of Veterinary Sciences of Turin within 1 h of collection.

### Blood culture

The samples were processed upon arrival at the laboratory. Briefly, each bottle underwent initial incubation at 36 ± 1° C for 1 h, followed by incubation with agitation (150 rpm) for 24 h. Then, the bottles were incubated without agitation for six days with daily observation. The presence of turbidity in the medium and/or fluid in the upper cylinder, indicates microbial activity. In such cases, subcultures were performed on blood agar (Oxoid, Basingstoke, UK) under both standard aerobic and anaerobic conditions. Identification of all bacterial colonies was performed using MALDI-TOF mass spectrometry (Bruker Daltonik GmbH, Bremen, Germany).

### 16S rRNA gene sequencing and data analysis

#### Blood fractions preparation.

From each calf, the first EDTA tube collected for 16S rRNA gene sequencing was divided into 500-µl aliquots for the whole blood fraction (**WB**). The second tube was centrifuged (3000 g x 15 min at 4° C) to separate the whole blood into fractions. Plasma (**PL**) was collected and centrifuged (8000 g x 5 min) at room temperature to pellet the remaining cells; the supernatant was used for analysis. The buffy coat (**BC**) phase was collected after removing the remaining plasma, and the red blood cell (**RBC**) fraction was obtained by pipetting from the bottom of the centrifuged tube. All samples were stored in DNA-RNA free Eppendorf and frozen at -80° C.

#### DNA extraction.

DNA was extracted from **WB**, **BC**, **RBC**, and **PL** fractions using a DNeasy Blood & Tissue Kit (Qiagen Ltd., Manchester, UK), according to the manufacturer’s instructions. DNA samples were eluted in 50 µL and stored at −20° C until further analysis. The DNA concentration of each sample was quantified using a Qubit 2.0 Fluorometer (ThermoFisher Scientific, Waltham, MA, USA) with the Qubit dsDNA HS Assay Kit (ThermoFisher Scientific). DNA integrity was evaluated by agarose gel electrophoresis (1% agarose in tris buffer EDTA 0.5X); DNA purity was determined by measuring the ratio of absorbance at 260/280 using a NanoDrop spectrophotometer (ThermoFisher Scientific). The samples meeting quality criteria were submitted for library preparation and metagenomic 16S rRNA gene sequencing.

#### Library preparation and sequencing.

DNA library preparation, 16S rRNA gene sequencing, and quality control were performed by Macrogen Europe (https://www.macrogen-europe.com/), a genomics service company. The library was prepared using Herculase II Fusion DNA Polymerase Nextera XT Index Kit V2 (Illumina, San Diego, CA, USA) following the 16S Metagenomic Sequencing Library Preparation Part # 15044223 Rev. B protocol. PCR amplification was performed to amplify the V3-V4 region of the 16S rRNA gene using the primers Bakt_341F: CCTACGGGNGGCWGCAG and Bakt_805R: GACTACHVGGGTATCTAATCC. The samples were run on an Illumina MiSeq platform to generate paired-end reads. The 16S rRNA gene sequencing raw data from Illumina sequencing comprised forward and reverse sequencing files. The sequences with high quality (quality score >20) were deemed suitable for further downstream analysis.

#### Bioinformatic analysis.

The fastq files were processed using QIIME2 (Quantitative Insights Into Microbial Ecology, release 2020.8) [24]. The DADA2 algorithm [25] (via q2-dada2 implementation) was used to filter the quality and to denoise the sequence data. In particular the forward and reverse reads were further cleaned by trimming off the first ten positions and truncating at positions 280 and 220, respectively, to remove low-quality portions.

The resulting amplicon sequence variants (ASVs) were cleaned from the chimeric sequences and taxonomically characterized by NCBI BLAST (Basic Local Alignment Search Tool, release 2.11.0) by aligning them on the NCBI 16S rRNA gene bacterial database (2023_09_30).

For each ASV, the best 20 alignments were selected and then filtered based on high levels of similarity (>= 99% identical matches, >= 99% query coverage, and eValue <0.05). This allowed one or more species to be associated with the ASVs characterized by at least one BLASTN result above the various cutoffs. Translation of GeneBank identifiers with Hugo Name is based on GeneGank db (2023_10_03). The abundance signal was further collapsed by summing the contributions of different ASVs attributed to the same species within each subject. α diversities were evaluated using the Chao1 Index, Shannon Index, and Inverse Simpson Index measures. Depending on the number of classes, their significance was analyzed using the Wilcoxon rank sum exact test or the Kruskal-Wallis test and subsequent pairwise comparison with the Wilcoxon rank sum exact test and Benjamini-Hockberg correction, when necessary.

β diversity was calculated with Bray-Curtis dissimilarity; its significance was analyzed using Permutational multivariate analysis of variance (PERMANOVA) based on 1000 permutations.

To mitigate the impact of contaminants and identify the bacteria linked to disease, the relative abundance of species in the samples from the **S** and the **H** subjects was compared. We utilized the modified Wilcoxon rank test (implemented through the ‘ziw’ function in the R package ZIR), specifically designed for Zero-Inflated Data (developed by Wanjie Wang, Eric Z. Chen, and Hongzhe Li in 2021). The resulting p-values were adjusted using the false discovery rate method (Benjamin-Hockberg correction). Statistical significance was set at alpha equal to 0.05, focusing exclusively on bacterial species that exhibited a significant increase in abundance in the samples associated to **S** calves. The final relative abundances of the significant species in each disease sample were calculated as follows: the median relative abundance observed in the **H** samples for each species was subtracted to each **S** sample, the resulting relative abundance for each species was recalculated to achieve a sum value of 1 in each disease sample.

### Statistical analysis

Statistical analysis was performed in R (release 4.3.2) and RStudio (release 2023.06.1 Build 524) environments. Normal distribution of data was assessed using the Shapiro-Wilk test. Data are expressed as mean and standard deviation (SD) or median and interquartile range (IQR) for continuous variables according to their distribution, and as percentages and frequencies for categorical nominal variables. The R packages used were: phyloseq (version 1.44.0), microbiome (version 1.22.0), vegan (version 2.6-4) and chvlyl/ZIR (version 1.0.0). Alpha was set at 0.05.

## Results

### Animals

The study sample was 18 Piedmontese calves, 8 of which were classified as **S** and 10 as **H**. Calves were mostly male (**S**: 7/8, 87.5%; **H**: 6/10, 60%) and no differences were found in the mean age between the groups (**S**: 10.75 ± 3.09 days; **H**: 11.10 ± 5.38 days).

### Clinical examination and blood culture

The **S** group calves were diagnosed with pneumonia (3/8, 37.5%), enteritis (3/8, 37.5%), and omphalitis (2/8, 25%). They had a probability of being bacteremic higher than 75%, based on the Fecteau score [22]. Blood cultures tested negative in 4/8 (50%); *Escherichia coli* (2/8, 25%), *Streptococcus uberis* (1/8, 12.5%)*,* and *Providencia stuartii* (1/8, 12.5%) were isolated from the positive samples.

### DNA extraction and quantification

For each animal, samples of the four matrices (**WB**, **PL**, **BC**, **RBC**) were sent for 16S rRNA gene analysis, therefore 72 samples were sent initially. The DNA was found suitable for sequencing according to the Macrogen Europe quality check in most of the samples (59/72, 82%): 35 samples were from the **H** group calves and 24 from the **S** group calves. The samples from the **H** group were mostly **WB** (10/35, 28.6%) and **PL** (10/35, 28.6%) fractions, while slightly fewer **BC** (8/35, 22.8%) and **RBC** (7/35, 20%) fractions. The samples from the **S** group were **PL** (8/24, 33.33%) and **WB** (7/24, 29.2), and **BC** fractions (6/24, 25%). Furthermore, only a few **RBC** fractions (3/24, 12.5%) could be submitted for analysis.

As shown in Table 2, for the **S** group, **RBC** had the maximum median value for the reads (2.87 x 10^5^, IQR: 2.46 x 10^5^ - 3.13 x 10^5^) and **WB** had the minimum median for the reads (1.66 x 10^5^, IQR: 1.48 x 10^5^–1.80 x10^5^). The **BC** values were significantly lower than those of the **RBC** (p < 0.05) or the **PL** (p ≤ 0.01); the **WB** fraction was lower than the **PL** fraction (p ≤ 0.01).

**Table 2:** Median reads for each blood fraction.

		Median (IQR) WB	Median (IQR) PL	Median (IQR) BC	Median (IQR) RBC
**Reads** **(x10**^**5**^)	**S**	1.66^a,c^	2.59^d^	1.80^d,c^	2.87^b^
		(1.48 - 1.80)	(2.26 - 3.05)	(1.64 - 1.93)	(2.46 - 3.13)
	**H**	1.65^e^	2.97^f^	1.71^f^	2.15^f^
		(1.58- 1.84)	(2.96 - 3.15)	(1.66 - 2.02)	(1.81 - 2.55)

**S:** suspected septic calves; **H:** healthy calves; **WB:** whole blood; **PL:** plasma; **BC:** buffy coat; **RBC:** red blood cells; **Different letters in the same row indicate a statistically significant difference: a, b** (p < 0.05), **c,d** (p ≤ 0.01)**; e,f** (p ≤ 0.001).

For the **H** group, the **PL** fraction had the maximum median for reads (2.97 x 10^5^, IQR: 2.96 x 10^5^–3.15 x 10^5^) and the **WB** fraction had the minimum median (1.65 x 10^5^, IQR 1.58 x 10^5^–1.84 x 10^5^). Significantly higher read values were noted for **PL** than for the other fractions (p ≤ 0.01). No significant differences were found when the same fractions were compared between the **S** and the **H** group.

### Microbiome analysis

**Fig 1:**
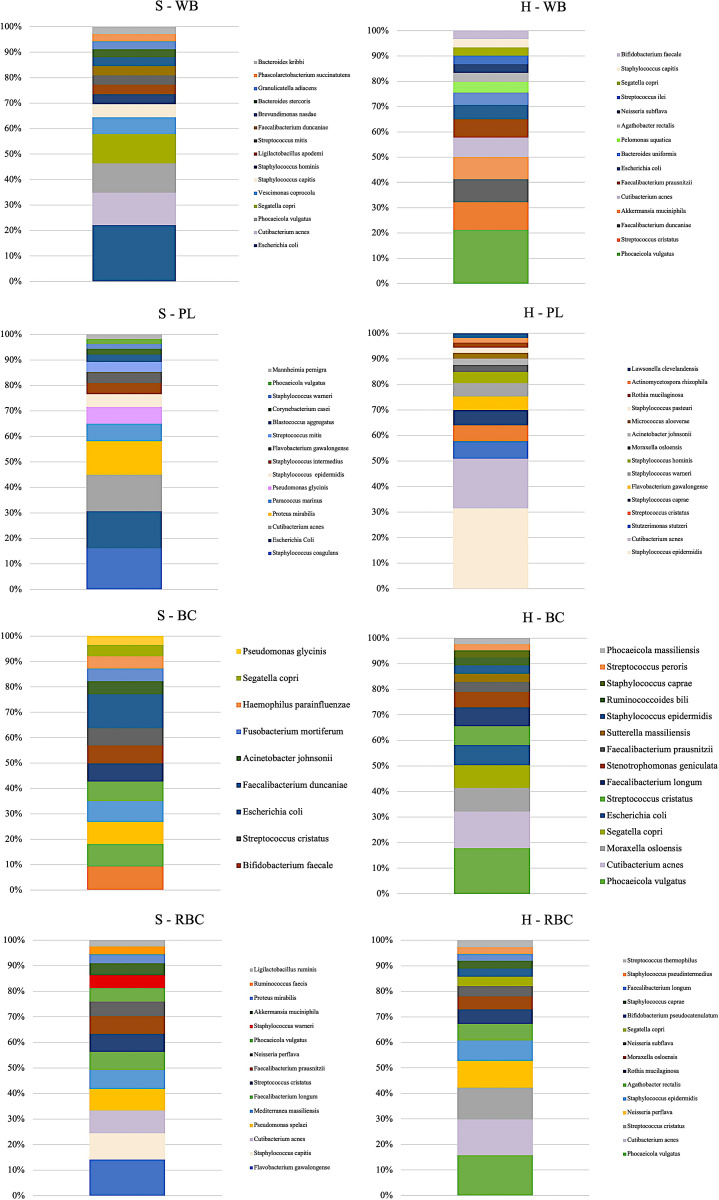
Median relative abundance (%) of the fifteen most abundant bacterial species identified in whole blood and each fraction of both groups. S: suspected septic calves; **H:** healthy calves; **WB:** whole blood; **PL:** plasma; **BC:** buffy coat; **RBC:** red blood cells.

**Fig 2:**
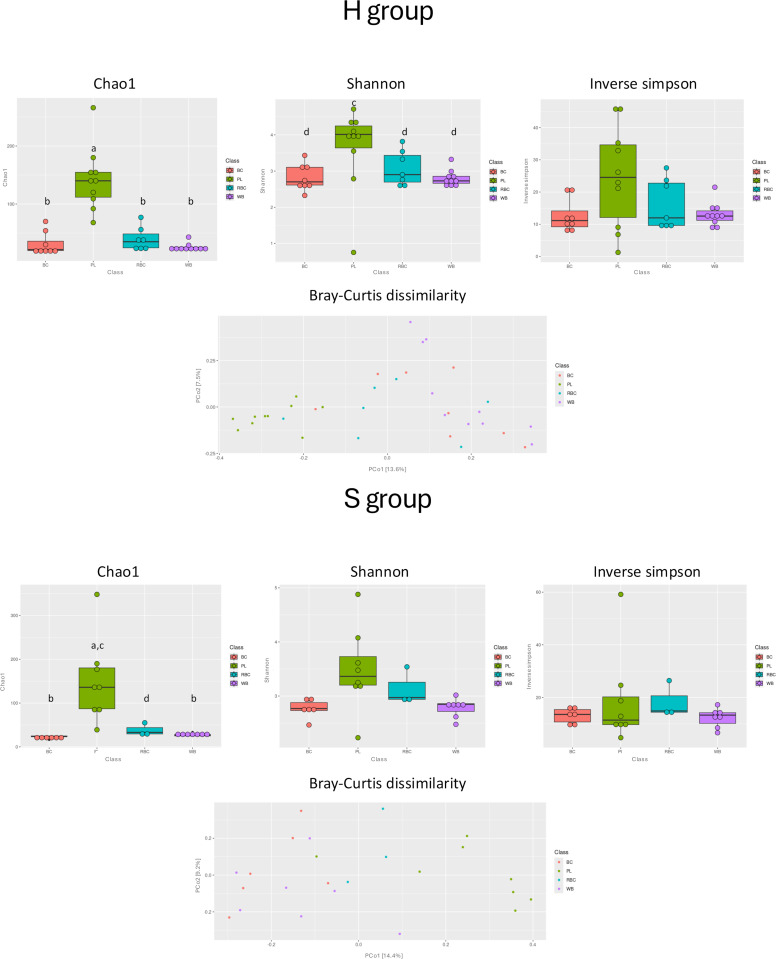
α and β diversity of blood fractions within S and H groups. S: denotes suspected septic calves; **H:** healthy calves; **WB:** whole blood; **PL:** plasma; **BC:** buffy coat; **RBC:** red blood cells; (**a,b:** p ≤ 0.01; **c,d:** p < 0.05).

The 15 most abundant microorganisms detected in each blood fraction are shown in Fig 1. For the **H** group, the most abundant bacterial DNA was *Phocaeicola vulgatus* detected in **WB**, **BC,** and **RBC,** while *Staphylococcus epidermidis* was most abundant in **PL**. For the **S** group, the most abundant bacterial DNA was *Escherichia coli* detected in **WB**, *Staphylococcus coagulans* in **PL**, *Bifidobacterium faecale* in **BC**, and *Flavobacterium gawaongense* in **RBC.** The α and β diversity indices differed across blood fractions within the same group of calves. In particular, the Chao1 (p ≤ 0.01) and the Shannon indices (p < 0.05) were significantly higher in the **PL** fraction compared to the other fractions from the **H** group. In the **S** group, the Chao1 was significantly higher in **PL** than in **RBC** (p < 0.05), **BC** or **WB** (p ≤ 0.01). β diversity analysis revealed statistically distinct microbial compositions in **PL** compared to the other fractions (Fig 2).

Comparison of the same blood fraction revealed no differences in the indices of α diversity between the **S** and the **H** group across all blood fractions, whereas significant differences between both groups were noted for the **PL** (p ≤ 0.01) and the **WB** (p < 0.05) fractions regarding β diversity analysis based on Bray-Curtis dissimilarity.

### Differential abundance analysis

**Fig 3:**
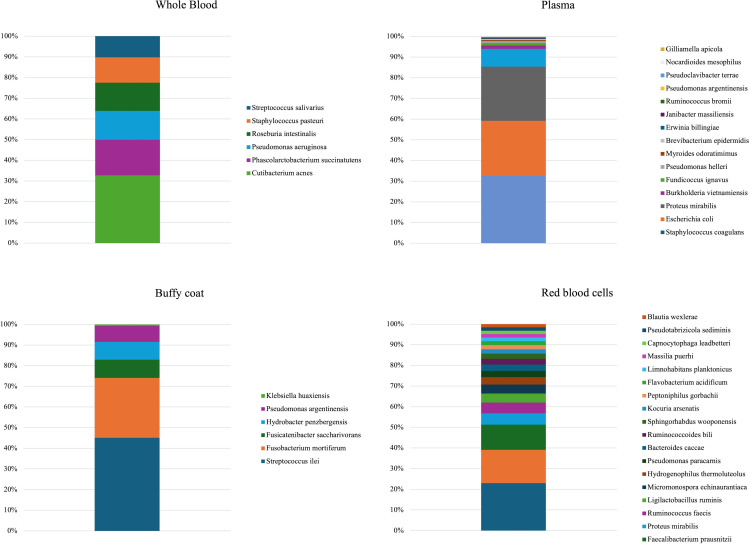
Median relative abundance (%) of bacterial DNA detected in different fractions in S group after differential abundance analysis.

**Fig 4:**
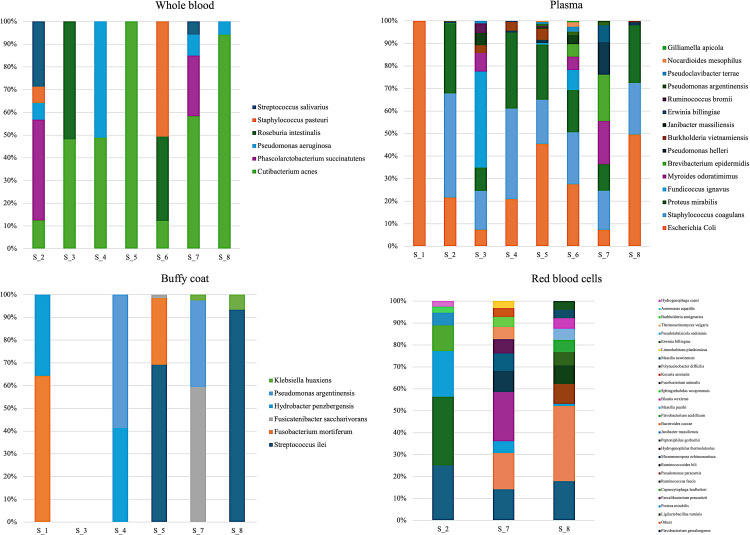
Relative abundance (%) of bacterial DNA detected in different fractions in each calf of S group after differential abundance analysis.

Differential abundance analysis revealed the presence of several bacterial species in the **S** group (Fig 3). The median relative abundance of *Cutibacterium acnes* DNA and *Phascolarctobacterium succinatutens* DNA were particularly high in **WB.** Considering the values of each calf, the DNA more frequently detected was from *Cutibacterium acnes* and *Pseudomonas aeruginosa* (Fig 4).

*Escherichia coli* DNA, *S. coagulans* DNA, and *Proteus mirabilis* DNA were particularly abundant in the **PL** fraction according to their median values and their abundance in each calf. *Streptococcus ilei* and *Fusobacterium mortiferum* DNA were more abundant in the **BC**. There was a variety of bacterial DNA in the **RBC** fraction, though their median relative abundance was generally low (Fig 3).

### Comparison between blood culture and differential abundance analysis

The bacterial species isolated from blood culture were *E.coli*, *S. uberis,* and *P. stuartii*. In the two **S** calves **(S_2, S_4)** in which *E. coli* was detected at blood culture, its bacterial DNA was significantly more abundant in the **PL** fraction than in that of the **H** calves (Fig 4). *E. coli* DNA was not significantly more abundant in the other blood fractions from either S_2 or S_4. Regarding S_2 specifically, P. succinatutens and *S. salivarius* DNA were significantly more abundant in the **WB** fraction, whereas *Ligilactobacillus ruminis* DNA and *Flavobacterium gawalongense* DNA were the most detected in **RBC**. For S_4, the most abundant bacterial DNA were *P. aeruginosa* and *C. acnes* in **WB,** while *Pseudomonas argentiniensis* and *Hydrobacter penzbergensis* DNA were most often detected in **BC** (Fig 4).

*S. uberis* was detected in S_6 at blood culture but not significantly more abundant than that in any blood fractions of the **H** group. The most abundant bacterial DNA detected in S_6 was *S. pasteuri* and *Roseburia intestinalis* in **WB***,* whereas *E. coli* and *S. coagulans* DNA was most often detected in **PL** (Fig 4).

*P. stuartii* was detected in S_7 at blood culture but its DNA was not significantly more abundant compared to **H** group. Most often detected in the **WB** of this calf was *C. acnes* and *P. succinatutens* DNA, whereas *Myroides odoratimimus* DNA and *Brevibacterium epidermidis* DNA were abundant in **PL** and *Fusicatenibacter saccharivorans* and *P. argentinensis* DNA were most often detected in **BC** (Fig 4).

## Discussion

To our best knowledge, this is the first study aimed to characterize and compare the bacterial DNA populations of whole blood and its fractions between healthy calves and those with suspected sepsis.

Analysis of the microbiome revealed bacterial DNA in whole blood and its fraction in both groups; nevertheless, understanding its origin and role poses challenges. The finding of bacterial DNA in the blood samples from the **S** calves may indicate an alteration of the physiological barrier between body districts, with bacteria entering the bloodstream [23,26,27]. Conversely, the presence of bacterial DNA in the blood samples from the **H** calves is more difficult to explain. Previous studies detected bacterial traces from blood culture or gene sequencing in healthy patients, but whether this should be considered a proper microbiome, sporadic bacteremia, cell-free DNA or environmental contamination remains debated [10,28–30]. Therefore, further studies are needed to fully understand the meaning of this finding.

Not all the samples were suitable for sequencing; indeed, most **RBC** fractions from the **S** group could not be analyzed. One possible explanation is that alterations in erythrocyte metabolism and shape during sepsis [31] may have reduced blood sample quality or perhaps this type of sample requires specific precautions for preservation and transportation before undergoing analysis.

Intra-group microbiome analysis showed differences for both the **S** and the **H** group. Differences between blood fractions have also been reported in human medicine [9]; to our knowledge, however, similar studies have not been conducted on cattle. Such differences may arise from the different mechanisms by which bacterial DNA enters the bloodstream. For example, bacterial DNA fragments can be detected in the plasma in health and diseased patients due to apoptosis and necrosis even when blood cultures are negative [32]. Further, phagocytosis by white blood cells can be responsible for detectable bacterial DNA within the **BC** fraction [9]. Also, it has been demonstrated that red blood cells can capture and transport bacterial DNA, which explains the bacterial DNA detected in the **RBC** fraction in our samples [33]. What is not completely clear, however, is whether such differences result from a distribution of bacterial DNA from other factors such as blood separation technique [14,34]. When we compared the same blood fractions between the two groups, the α diversity indices indicated no significant differences, whereas Bray-Curtis dissimilarity disclosed differences between both groups for the **PL** and the **WB** fraction. Differential abundance analysis also showed significant differences. The relative abundance of the DNA of several bacterial species was statistically higher in the **S** than in the **H** group (Figs 3 and 4). It is possible that due to infection and inflammation bacteria entered the bloodstream from other body sites [35,36]. The higher abundance of *P. aeruginosa* DNA in the **WB** fraction from the **S** group could imply its role in sepsis, as it’s a well-known opportunistic bacterium [37].

In this fraction, there was also a high relative abundance of *Cutibacterium acnes*, *Phascolabacterium sucinatutens*, *Roseburia intestinalis*, and *Staphylococcus. pasteuri* DNA. *Cutibacterium acnes*, previously known as *Propionibcterium acnes*, a common inhabitant of the human skin and associated with pathological conditions [38], has been detected in the blood of septic human patients [13,39]. Moreover, since other studies found it in the rumen, placenta, and cow brain [40–42], we cannot exclude its involvement in the sepsis in the **S** calves. *S. pasteuri* has been detected in udders with mastitis [43], while the role of *Roseburia intestinalis* and *Phascolabacterium succinatutens* is unclear. The former appears to have a limiting effect on bacterial translocation owing to its butyrate production. Previous studies have reported it in cow gut, milk, and vaginal microbiomes [44–46]. The latter is an inhabitant of the ruminant digestive system and uses succinate for growth [47]. Therefore, their presence in the bloodstream could indicate bacterial translocation from the gut.

The *E. coli* DNA in the **PL** fraction was significantly more abundant in the **S** group. *E. coli* is a well-known microorganism in calf septicemia and our findings indicate that **PL** may be more efficient than the other blood fractions for detecting the DNA of this microorganism [48]. The relative abundance in **PL** of *S. coagulans* DNA was also high. This bacterium inhabits the mucous membranes and skin and has been associated with mastitis in some cases [49].

Though not commonly known to be pathogenic, the bacterial DNA of *S. ilei* and *Fusobacterium mortiferum* was significantly more often found in the **BC** fraction of the **S** group*. S. ilei* is a recently discovered microorganism [50] in humans. Further studies are needed to understand its role in cattle. *Fusobacterium mortiferum* has been isolated in cows with digital dermatitis and in the rumen [51,52]. Its presence in our sample might be attributed to bacterial translocation.

Several microorganisms had a higher relative abundance in the **RBC** fraction in the **S** group. Aside from the detection of the DNA of the potentially pathogenic bacterium *Proteus mirabilis*, the other bacterial DNA detected in RBC are opportunistic microorganisms are rarely studied in cattle [53,54]. However, because **RBC** could be analyzed for only three animals, a larger sample is needed to understand the role of these bacteria in this fraction.

The sequencing data indicate that the abundance of bacterial DNA from different species can differ in whole blood and its fractions. In our study, pathogenic bacteria (e.g., *E. coli)* was more often found in the **PL** than in the other fractions or whole blood. Nevertheless, analysis of **PL** alone would results in a loss of data on bacterial DNA in the bloodstream. While the underlying reasons for this variability remain unclear, our findings are shared by previous studies on humans which did not clarify which blood fraction can give a comprehensive overview of bacterial DNA in the bloodstream. [13,32,55].

*E. coli* was detected by blood culture in only two calves, while its DNA was significantly more abundant in the **PL** of 8 **S** calves compared to the **H** group. The origin of bacterial DNA in **PL** warrants further study, therefore our findings are insufficient to indicate whether viable *E. coli* was present even when the blood culture was negative. Studies in human medicine suggest, however, that the microbial DNA levels detected at sequencing may reflect the dynamics of infection [32,56], even when the microorganism is not detected by culture, and therefore could indicate a role for *E. coli* in clinical status. Further studies are needed to understand whether analysis of **PL** could help to evaluate the infection dynamics.

Several uncommon pathogens were isolated at blood culture in two calves. *S. uberis* is a relatively uncommon environmental pathogen affecting calves. Its involvement in sepsis should not be underestimated since it’s related to udder diseases and has also been detected in the blood of adult cows [36,57]. Similarly, *P. stuartii* is an environmental microorganism that inhabits cattle intestines; its detection in blood is unusual [58]. In human medicine, it has been detected in septic patients [59]. In our sample, the relative abundance did not significant differ between the **S** and **H** groups; therefore, we believe these bacteria might have been contaminants.

Despite we applied the commonly described techniques to perform sterile blood draws, it must be considered that there is a lack of literature describing the optimal procedures for performing a sterile blood sampling, in bovine, on field condition [8]. Pas et al. [8] reported a 14.90% contamination rate in blood cultures from calves although the sampling site had been prepped. While skin disinfection reduces the microbial population originating from cow hair and skin [60,61], routine procedures may not be sufficient to prevent contamination of blood cultures. In humans, repeated surgical site preparation before surgery was found to reduce skin bacterial loads [62]. Given the difficulty of maintaining a proper asepsis during on-field sampling, further studies should evaluate the effectiveness of a second surgical scrub, performed between the two sampling, on limiting the contamination of blood culture. Other factors were likely also at play in the differences between the blood culture results and the 16S rRNA gene sequencing analysis. Indeed, both clinical evaluation and blood culture have their limitations. Currently available clinical evaluation scores are imperfect tools for detecting sepsis in ruminants. The SIRS score can be influenced by the behavioral response of the patient [2,63]. In human medicine, the sepsis-related organ failure assessment (SOFA) score [4] appears efficient in the clinical evaluation of sepsis; however, it entails assessment of multiple parameters, such as fraction of inspired oxygen or arterial pressure, which are difficult to measure in buiatric practice. Moreover, this score has not yet been validated in ruminants. In the present study we sought to overcome the limitations of the SIRS score by including only critically ill calves [1]. The bacteremia score has a sensitivity and a specificity of 75% and 71%, respectively [22], which may explain why some calves tested negative at blood culture despite having a high score.

Blood culture has its limitations, too [5,6]. A low bacterial load can produce a negative culture. 16S rRNA sequencing has drawbacks as well: it does not discern between live and dead microorganisms nor it allow for antimicrobial susceptibility testing, which could be crucial for determining therapy effectiveness and assessing the antimicrobial resistance status of a herd.

None of these techniques is flawless and sepsis is not always associated with bacteremia. Indeed, the presence of cell-free bacterial DNA, detectable at 16S gene sequencing but not with culture-based methods, can trigger immune response in some cases [64]. If we wish to gain a deeper understanding of the mechanisms underlying sepsis, we will need to overcome the limitations of current diagnostic techniques. Lastly, not all the calves had the same site of infection. Owing to the small sample size, we were unable to analyze the effect of infection on the blood microbiome. Nevertheless, sepsis caused a shift in the bacterial DNA towards microorganisms with potential pathogenicity in the **S** calf group. Further, our findings are shared by studies in human medicine that suggest that the microbial population tends to shift during sepsis, regardless of whether there is one or multiple infection sites [12,65,66].

In conclusion, 16S rRNA gene sequencing holds promise for the evaluation of bacterial DNA in the blood fractions of **S** calves. The difference in the microbial population between **PL** and the other blood fractions suggests that **PL** may be a useful matrix for monitoring infection dynamics during sepsis. Further studies with larger samples are needed to better understand the role of bacteria in the bloodstream.

## Supporting information

S1 Dataset for the analysis. (XLSX)
